# A novel prognostic index—neutrophil times γ-glutamyl transpeptidase to lymphocyte ratio (NγLR) predicts outcome for patients with hepatocellular carcinoma

**DOI:** 10.1038/s41598-017-09696-y

**Published:** 2017-08-23

**Authors:** Jun Li, Yan Liao, Liya Suo, Pengpeng Zhu, Xinhuang Chen, Wei Dang, Minjun Liao, Liling Qin, Weijia Liao

**Affiliations:** 1 Laboratory of Hepatobiliary and Pancreatic Surgery, Affiliated Hospital of Guilin Medical University, Guilin, 541001 Guangxi People’s Republic of China; 2 Disease Prevention and Control Center of Guilin, Guilin, 541001 Guangxi People’s Republic of China; 3 Guangxi Medical University, Nanning, 530021 Guangxi People’s Republic of China

## Abstract

Clinical outcomes of patients with hepatocellular carcinoma (HCC) are highly variable. This study aims to identify and validate a simple, readily available, and objective prognostic index for the management of HCC. Data from 724 HCC patients undergoing curative resection were evaluated and randomly divided into two cohorts for building and validating the prognostic index. A best model, NγLR = (neutrophil count [10^9^/L] × γ-glutamyl transpeptidase [U/L]) /(lymphocyte count [10^9^/L] × U/L), was selected. An optimal cut-off value of 103.6 for NγLR stratified patients into high NγLR (>103.6) and low NγLR (≤103.6) groups. NγLR > 103.6 was closely associated with HCC malignant characteristics. Elevated NγLR predicted a worse overall survival (OS) and progression-free survival (PFS) for HCC patients and remained an independent predictor for both types of survival. Moreover, early recurrence rates in patients with NγLR > 103.6 were higher than that in patients with NγLR ≤ 103.6 (*P* < 0.0001). NγLR was an important independent predictor of survival for HCC patients and might be a new promising method to identify patients at different risks of early recurrence and survival after curative resection.

## Introduction

Hepatocellular carcinoma (HCC) is one of the most common cancers worldwide, with approximately 745,000 deaths each year^1^. Unlike other cancers, the clinical manifestations of HCC are remarkably heterogeneous, and the prognosis of HCC is also complex and multifaceted, leading it to be the third most lethal malignant tumour worldwide^2^. Despite the advances in diagnostic and therapeutic methods, the 5-year risk of recurrence of HCC after surgery is as high as 70%^3^. A high incidence of HCC exists in Southeast Asia and sub-Saharan Africa, where the infection of hepatitis B virus (HBV) is endemic, while the incidence of HCC in western countries has also increased in recent years^2, 4^. In the United States, the incidence rate of HCC more than doubled between 1985 and 2002, due to chronic hepatitis C virus infection, alcohol-related cirrhosis, or possibly the prevalence of obesity and diabetes^2^.

It is clear that the high recurrence rate of HCC after surgery is a major obstacle faced by clinicians. In recent decades, a wide range of prognostic staging systems, including the Cancer of the Liver Italian Program^5^, Barcelona Clinic Liver Cancer (BCLC), Tumour-Node-Metastasis (TNM), the Chinese University Prognostic Index^6^, the Tokyo scoring system^7^, and the Hong Kong Liver Cancer staging system^8^, have been proposed, among others. In general, these prognostic models have unique prognostic performance, but none of them is universally accepted^9^. Numerous efforts have been devoted to identify molecular signatures to guide prognosis prediction in HCC patients, such as microRNA, gene signatures, and epigenetic biomarkers^10–12^. However, gene expression analysis is expensive and highly sophisticated, limiting its applicability to patients in routine clinical practice.

Recently, in the setting of precision medicine, researchers have been trying to find a potential marker or develop a multi-factor model that can predict the risk and prognosis of HCC from the peripheral blood early in the course of the disease. For example, a simple risk score composed of routinely measured parameters was constructed to predict the incidence of HCC in HBV carriers^13^; a simple model based solely on serum bilirubin and albumin levels was developed to assess liver function in patients with HCC^14^; an elevated neutrophil-lymphocyte ratio predicted adverse outcomes for HCC patients after living-donor liver transplantation^15^; a systemic immune-inflammation index [SII] score >330 was related to a higher recurrence rate and a shorter survival in patients with HCC^16^; and the aspartate transaminase (AST) to lymphocyte ratio index (ALRI) was built to predict different prognoses among various subgroups of HCC^17^.

For most clinicians, a simple, objective, and readily-calculated model is preferred. In this study, we identified a novel and simple laboratory index, i.e. neutrophil times γ-glutamyl transpeptidase to lymphocyte ratio (NγLR) in large cohorts of patients, and then validated its diagnostic accuracy in another independent cohort. We found the prediction ability of the NγLR to be generally high throughout the training cohort. The correlation between the NγLR and clinicopathologic parameters was also explored, and the impact of an elevated NγLR on postoperative survival and recurrence was systematically evaluated.

## Materials and Methods

### Patients

**Figure 1:**
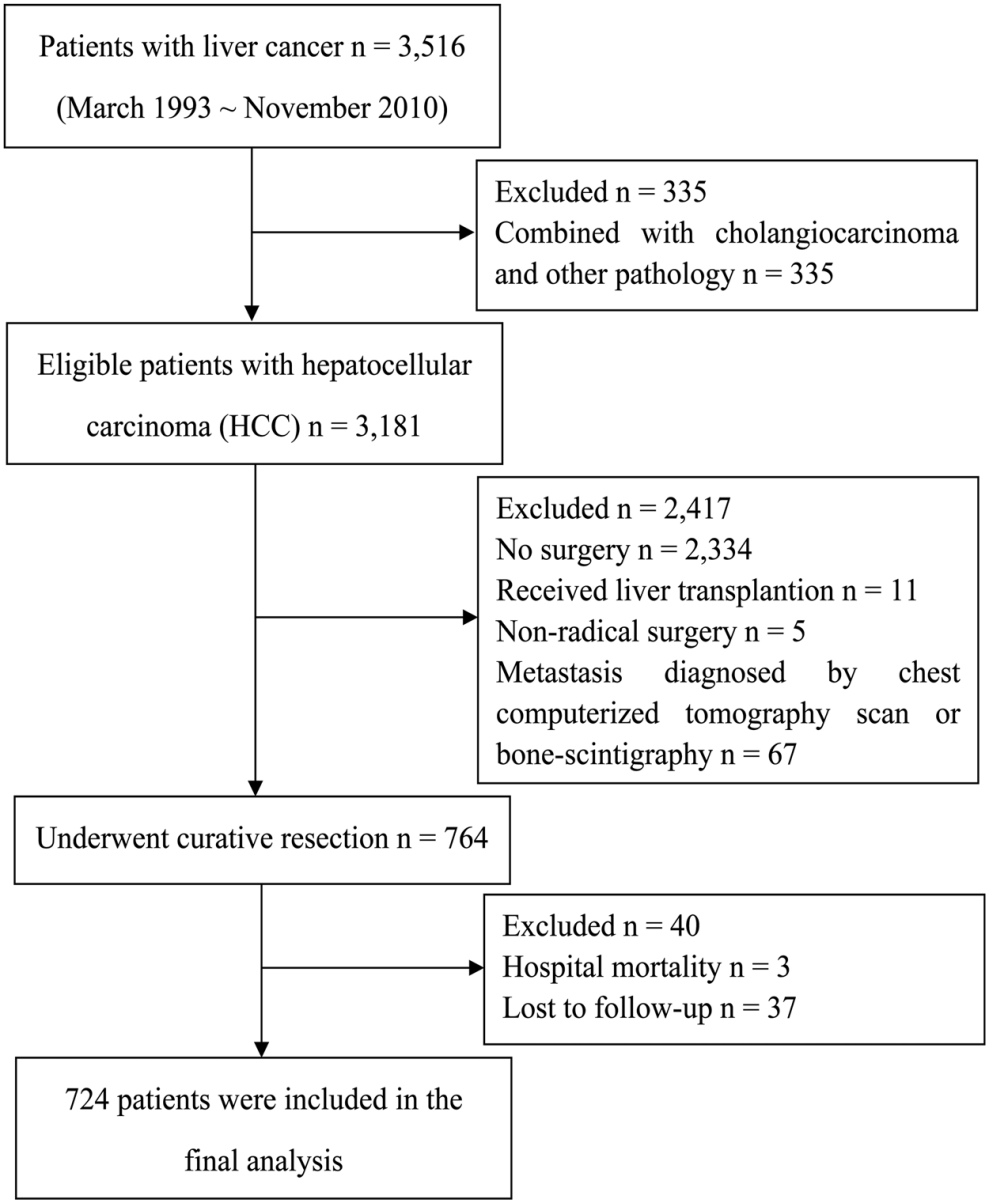
Flow diagram of patients enrolled in this study and reasons for exclusion.

Between March 1993 and November 2010, 3,516 patients newly diagnosed with liver cancer at the Affiliated Hospital of Guilin Medical University (Guilin, People’s Republic of China) were evaluated retrospectively. In the light of the included and excluded criteria, a total of 724 patients who underwent curative resection for HCC were eligible for this study (Fig. 1). The diagnostic criteria of HCC were based on clinical, serological, ultrasonography (US), computerized tomography (CT), magnetic resonance imaging (MRI), and pathologic examinations according to the Primary Liver Cancer Clinical Diagnosis and Staging Criteria (Ministry of Health, Beijing, China). Curative resection was defined as a complete resection of the tumour, a resection margin of at least 1 cm, no residual tumour based on histological examination, and no residual tumours or new lesions determined by two observations not less than 4 weeks apart^18^. Both intraoperative US and postoperative CT were used to confirm complete removal of HCC. The baseline information including demographic characteristics, hepatitis B surface antigen (HBsAg), hepatitis C virus antibody, use of nucleoside analogues (NAs), presence of liver cirrhosis, complete blood count, albumin, globulin, total bilirubin, direct bilirubin, alanine aminotransferase (ALT), AST, alpha-fetoprotein (AFP), γ-glutamyl transpeptidase (γ-GT), tumour characteristics, metastasis and recurrence were collected. All laboratory parameters used in the study were measured before curative resection. All methods were carried out in accordance with Affiliated Hospital of Guilin Medical University guidelines and regulations. This study was approved by the research ethics committee of Affiliated Hospital of Guilin Medical University and complied with The Declaration of Helsinki Principles. Informed consent was obtained from all patients.

### Follow-up and postoperative treatment

All postoperative patients were followed with the regular monitoring of serum AFP concentrations and abdomen US every 2 months and chest radiography every 6 months during the first two postoperative years and at 3–6 month intervals thereafter. Further examinations, including CT and MRI, were performed if recurrence or metastasis was suspected. When recurrence or metastasis was confirmed, further treatment, i.e., a second surgical resection, radiofrequency ablation, transcatheter arterial chemoembolization, percutaneous ethanol injection or sorafenib treatment were suggested. The main clinical endpoint of this study was overall survival (OS), measured from the date of surgery to the date of death or the last follow-up. Progression-free survival (PFS) was calculated from the date of surgery to the date of recurrence, metastasis, death, or last follow-up. The cut-off value between early and late recurrence was set as 2 years.

### Statistical methods

All 724 patients were randomly assigned to either a training cohort for developing a new prognostic index or a validation cohort for evaluating the obtained prognostic index at an approximately 2:1 ratio. Variables were expressed as the mean ± SD unless otherwise stated. Categorical data were compared by the Pearson χ^2^ test or the Fisher exact test, while continuous variables were assessed by Student’s t test. OS and PFS were calculated using the Kaplan–Meier method and compared with the log-rank test. Univariate analysis was performed to identify significant prognostic factors by using the Cox regression model. The multivariate Cox proportional hazards regression model was performed to identify independent predictors by including all the variables demonstrated to be significant in the univariate analyses. All statistical analyses were performed with SPSS18.0 (SPSS Inc., Chicago, IL). Statistical significance was defined as *P* < 0.05.

### Derivation of a prognostic model

To construct a best prognostic model, an exploratory formula was built by entering different sets of the independent factors into a regression model. The best model based on the preoperative peripheral blood neutrophil count, lymphocyte count, and serum γ-GT level was selected from the training cohort. The calculation formula for the novel prognostic model was as follows: NγLR = (neutrophil count [10^9^/L] × γ-GT [U/L])/(lymphocyte count [10^9^/L] × U/L). The diagnostic accuracy of the new index was estimated with the receiver operating characteristic (ROC) curve. Optimal cut-offs for NγLR were selected in terms of maximizing both the sensitivity and specificity. The ROC curves were compared between different prognostic models for HCC patients using the area under the ROC curves (AUC). Finally, the performance of the NγLR index was tested in the validation cohort.

### Data availability

The datasets generated during and/or analysed during the current study are available from the corresponding author on reasonable request.

## Results

### Characteristics of the patients with HCC

Seven hundred and twenty-four HCC patients, with 463 in the training cohort and 261 in the validation cohort, were enrolled in this study. The clinical and laboratory characteristics of the HCC patients in both training and validation cohorts are shown in Table 1. Overall, the clinicopathologic characteristics between these two cohorts were very similar, except for smoking. The mean age of the patients in the training cohort was 50.14 ± 11.58 years and 49.56 ± 10.84 years in the validation cohort. In both training and validation cohorts, most patients had hepatitis B-related liver disease (85.3% and 82.8%, respectively) and cirrhosis (93.5% and 93.5%, respectively), and the numbers of HBsAg positive patients under NAs were 90 cases and 44 cases, respectively. Microvascular invasion was present in 17.7% of patients in the training cohort and 23.4% in the validation cohort. The median OS times were 47.12 (95% CI, 44.37–50.07) and 51.02 (95% CI, 47.23–54.71) months in the training and validation cohorts, respectively, and the median PFS times were 39.83 (95% CI, 36.78–42.91) and 40.27 (95% CI, 36.29–44.37), respectively. A total of 185 (39.96%) patients in the training cohort and 109 (41.76%) patients in the validation cohort experienced recurrence by the time of the data analysis.

**Table 1:** Clinical and biochemical data of examined patients.

Parameter	Training cohort	Validation cohort	*P* value
	(n = 463)	(n = 261)	
Age (years)	50.14 ± 11.58	49.56 ± 10.84	0.504
Gender: female/male (n)	62/401	38/223	0.662
Family history: absent/present (n)	400/63	220/41	0.439
Drinking: absent/present (n)	245/218	153/108	0.138
Smoking: absent/present (n)	249/214	167/94	0.008
Cirrhosis: absent/present (n)	30/433	17/244	0.986
HBsAg: negative/positive (n)	68/395	45/216	0.363
HCVAb: negative/positive (n)	454/9	251/10	0.127
MaI: absent/present (n)	381/82	200/61	0.066
NγLR: median, range	137.54, 15.02–1507.96	133.59, 16.73–1236.02	0.746
SII: median, range	327.63, 5.72–2260.07	318.50, 4.35–2168.96	0.833
ALRI: median, range	24.65, 2.47–417.12	23.31, 0.94–320.96	0.540
WBC (×10^9^/L)	6.15 ± 1.99	6.23 ± 2.00	0.598
NEUT (×10^9^/L)	3.74 ± 1.70	3.80 ± 1.64	0.677
LYMPH (×10^9^/L)	1.70 ± 0.63	1.72 ± 0.58	0.685
Platelets (×10^9^/L)	175.62 ± 79.28	185.04 ± 82.63	0.131
Albumin (g/L)	39.17 ± 4.46	39.13 ± 4.34	0.915
Globulin (g/L)	30.87 ± 5.29	31.21 ± 4.96	0.388
TBIL (μmol/L)	17.82 ± 33.40	18.86 ± 41.06	0.719
DBIL (μmol/L)	7.94 ± 24.72	8.13 ± 25.05	0.850
ALT (U/L)	47.55 ± 43.51	45.39 ± 46.75	0.369
AST (U/L)	54.22 ± 55.06	53.69 ± 57.51	0.801
AFP (ng/ml): median, range	152.33, 0.23–11602.00	161.25, 0.61–10340.00	0.549
γ-GT (U/L): median, range	68.60, 10.00–657.30	67.50, 15.10–604.90	0.902
Use of NAs: absent/present (n)	310/93	181/44	0.306
Tumor size (cm)	7.82 ± 4.77	7.43 ± 4.65	0.183
Tumor number: single/multiple (n)	327/136	200/61	0.081
Tumor differentiation: I/II/III/IV (n)	61/180/178/44	43/89/91/38	0.085
Child-Pugh classification: A/B (n)	425/38	237/24	0.648
BCLC stage: 0/A/B/C (n)	20/232/116/95	18/127/78/38	0.076
Type of surgical resection^a^ (Anatomical/non-anatomical)	282/181	165/96	0.539

^a^Anatomical resection includes hemihepatectomy, sectorectomy, and segmentectomy; non-anatomical resection includes limited resection and tumor enucleation. n, number of patients; HBsAg, hepatitis B surface antigen; HCVAb, hepatitis C virus antibody; MaI, microvascular invasion; NγLR, neutrophil cell count times γ-glutamyl transpeptidase to lymphocyte count ratio; SII, systemic immune-inflammation index; ALRI, aspartate transaminase to lymphocyte ratio index; WBC, white blood cell; LYMPH, lymphocyte count; TBIL, total bilirubin; DBIL, direct bilirubin; ALT, alanine aminotransferase; AST, aspartate aminotransferase; AFP, alpha-fetoprotein; γ-GT, γ-glutamyl transpeptidase; NAs, nucleoside analogues; BCLC, barcelona-clinic liver cancer.

### Comparisons of AUC between NγLR and other prognostic indices

**Figure 2:**
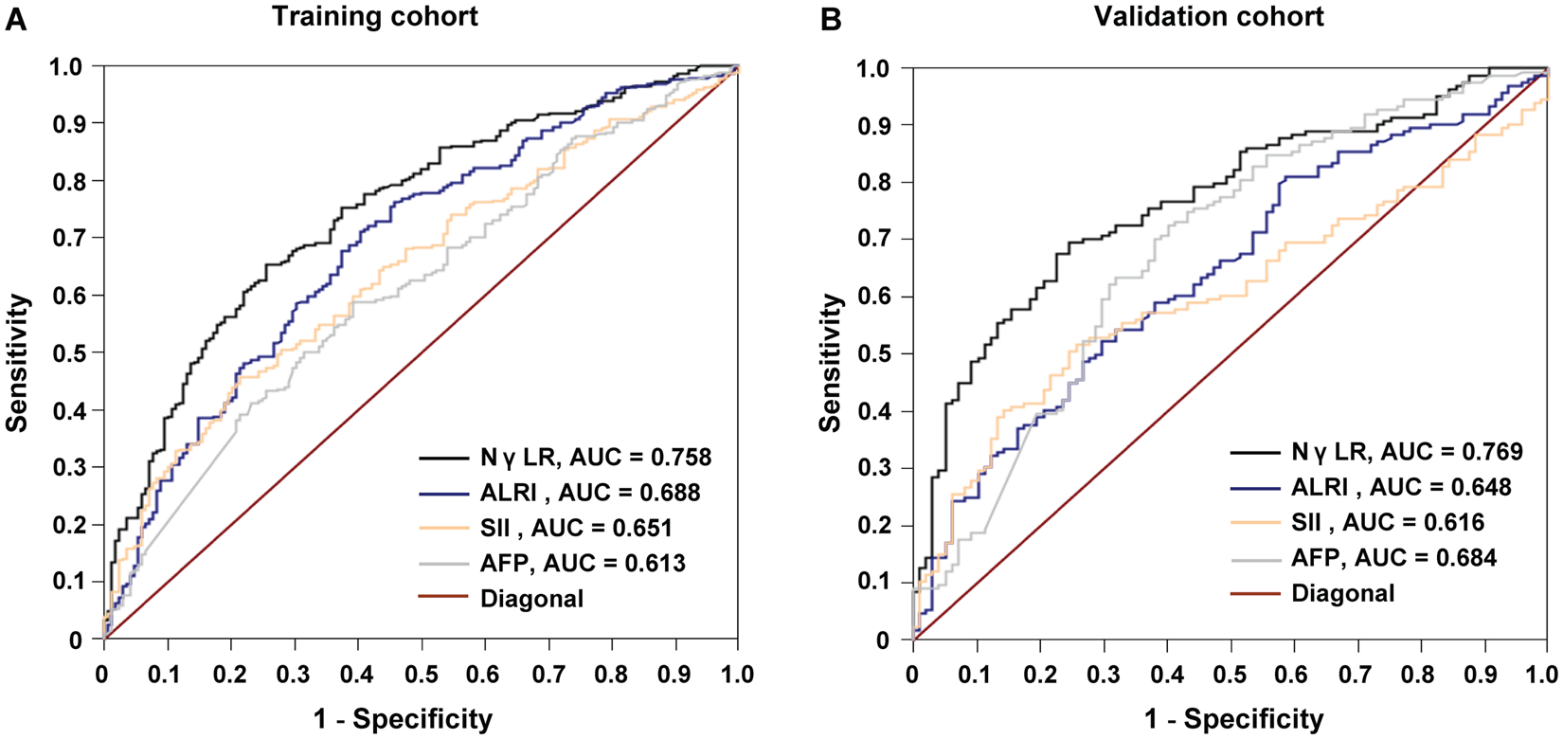
Predictive ability of the NγLR and the correlation between NγLR and AFP. The predictive ability of NγLR was compared with other prognostic parameters by ROC curves in the training (**A**) and the validation (**B**) cohorts.

Several useful prognostic models resulted from previous research, such as SII and ALRI^16, 17^. Therefore, we used the thresholds of these models to assess the prognosis of the HCC patients selected in the present study. The prediction abilities of the NγLR, SII, ALRI and conventional parameters, such as AFP, were compared. In the training cohort, the AUC for the NγLR was 0.758 (95% CI, 0.714–804), which was higher than those of SII (0.651, 95% CI, 0.602–0.703), ALRI (0.688, 95% CI, 0.641–0.738) and AFP (0.613, 95% CI, 0.562–0.665) (Fig. 2A).

The discrimination ability of NγLR was further confirmed in the validation cohort. For predicting OS, the AUC of NγLR index (0.769, 95% CI, 0.712–0.823) was significantly better than those of SII (0.616, 95% CI, 0.549–0.692), ALRI (0.648, 95% CI, 0.583–0.719) and AFP (0.684, 95% CI, 0.620–749) (Fig. 2B).

### Association between NγLR and clinicopathologic parameters

On the basis of the AUC analysis, an optimal cut-off value of the NγLR to stratify patients between high and low survival for postoperative patients was 103.6 in the training cohort. Subsequently, the NγLR was stratified into ≤103.6 or >103.6 for all subsequent analyses. In the training cohort, NγLR > 103.6 was positively correlated with gender (*P* = 0.006), a tumour size >8 cm (*P* < 0.001), multiple tumour number (*P* = 0.003), poor tumour differentiation (*P* < 0.001), vascular invasion (*P* < 0.001), BCLC stage B and C (*P* < 0.001), early recurrence (*P* = 0.008), AFP (>20 ng/ml) (*P* = 0.035), ALT (>40 U/L) (*P* < 0.001), SII (>330) (*P* < 0.001), ALRI (>25.2) (*P* < 0.001), and use of NAs (*P* < 0.001) (Table 2). The relationships between NγLR and the clinicopathologic parameters in the validation cohort were very similar to those in the training cohort (Table 2).

**Table 2:** Correlation between the clinicopathologic variables and NγLR level in HCC patients (training cohort, n = 463 and validation cohort, n = 261). NγLR, neutrophil cell count times γ-glutamyl transpeptidase to lymphocyte count ratio; HBsAg, hepatitis B surface antigen; BCLC, barcelona-clinic liver cancer; AFP, alpha-fetoprotein; ALT, alanine aminotransferase; SII, systemic immune-inflammation index; ALRI, aspartate transaminase to lymphocyte ratio index; NAs, nucleoside analogues.

Variables		NγLR level					
		Training cohort			Validation cohort		
		≤103.6	>103.6	*P* value	≤103.6	>103.6	*P* value
Gender	Female	32	30	0.006	21	17	0.020
	Male	135	266		79	144	
Age (years)	≤55	107	200	0.445	63	114	0.189
	>55	60	96		37	47	
HBsAg	Negative	24	44	0.885	22	23	0.109
	Positive	143	252		78	138	
Tumor size	≤8 cm	145	149	<0.001	94	93	<0.001
	>8 cm	22	147		6	68	
Tumor number	Single	132	195	0.003	84	116	0.027
	Multiple	35	101		16	45	
Tumor differentiation	I- II	132	109	<0.001	78	54	<0.001
	III- IV	35	187		22	107	
Vascular invasion	Absent	158	223	<0.001	92	108	<0.001
	Present	9	73		8	53	
BCLC stage	0 + A	125	127	<0.001	76	69	<0.001
	B + C	42	169		24	92	
Early recurrence	Absent	135	206	0.008	62	120	0.032
	Present	32	90		38	41	
AFP (ng/ml)	≤20	59	77	0.035	46	46	0.004
	>20	108	219		54	115	
ALT (U/L)	≤40	122	103	<0.001	72	67	<0.001
	>40	45	193		28	94	
SII (×10^9^/L)	≤330	122	101	<0.001	62	52	<0.001
	>330	45	195		38	109	
ALRI	≤25.2	129	103	<0.001	69	75	<0.001
	>25.2	38	193		31	86	
Use of NAs	Absent	89	221	<0.001	58	123	0.012
	Present	45	48		23	21	

### Prognostic factors and survival in the training cohort

**Figure 3:**
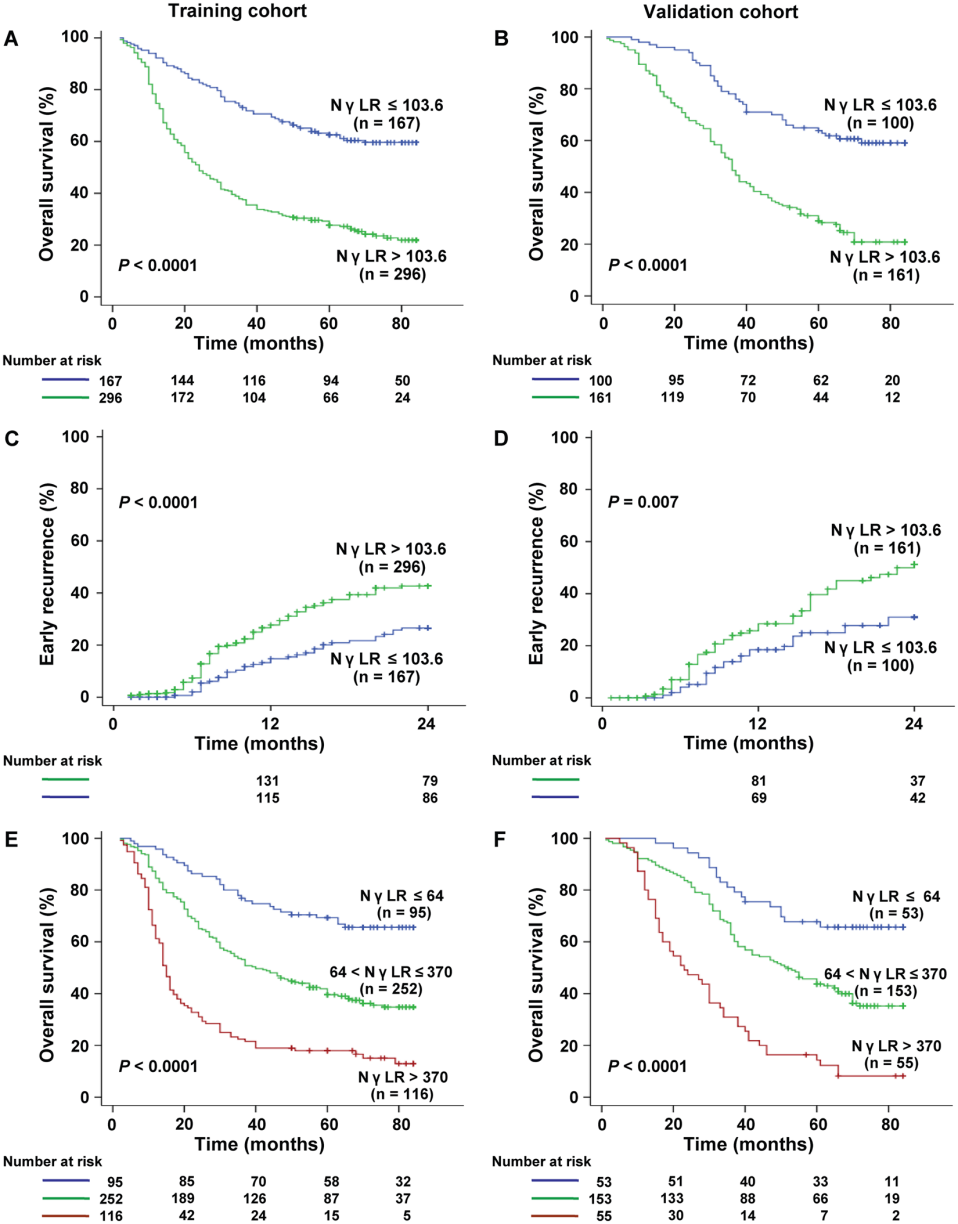
Prognostic significance of NγLR in patients with HCC after surgery. The Kaplan-Meier curves depict overall survival (**A,B**) and recurrence rates (**C,D**) in HCC patients with NγLR > 103.6 or ≤103.6 and estimate the overall survival according to the optimized NγLR (E-F) in the training and validation cohorts.

In the univariate analyses, a tumour size of >8 cm, multiple tumour number, poor tumour differentiation, vascular invasion, BCLC stage B and C, AFP (>20 ng/ml), ALT (>40 U/L), SII (>330), ALRI (>25.2), and NγLR (>103.6) were identified as significant prognostic factors of poor OS and PFS, whereas received NAs as antiviral treatment was associated with better OS and PFS in the training cohort (all *P* < 0.05) (Table 3 and Supplementary Table S1). The Kaplan–Meier analysis showed that a high NγLR was associated with a shorter OS (*P* < 0.0001) and PFS (*P* < 0.0001) (Fig. 3A and Supplementary Fig. S1A). The median OS and PFS times were 62.31 (95% CI, 57.94–66.67) and 56.02 (95% CI, 50.75–61.29) months for patients with NγLR ≤ 103.6, compared with 37.25 (95% CI, 33.86–40.64) and 31.48 (95% CI, 27.95–35.01) months for patients with NγLR > 103.6. The 1-, 3-, and 5-year OS rates were significantly lower in the subgroup with NγLR > 103.6 (78.68%, 37.12%, and 27.25%, respectively) than in the NγLR ≤ 103.6 group (94.12%, 73.02%, and 62.24%, respectively) (*P* < 0.0001).

**Table 3:** Analysis of overall survival in HCC patients in the training and validation cohort. HBsAg, hepatitis B surface antigen; BCLC, barcelona-clinic liver cancer; AFP, alpha-fetoprotein; ALT, alanine aminotransferase; SII, systemic immune-inflammation index; ALRI, aspartate transaminase to lymphocyte ratio index; NγLR, neutrophil cell count times γ-glutamyl transpeptidase to lymphocyte count ratio; NAs, nucleoside analogues.

Variable	Univariate analysis			Multivariate analysis		
	HR	95% CI	*P* value	HR	95% CI	*P* value
*Training cohort*						
Gender (male *vs* female)	1.26	0.89–1.76	0.092			
Age, y (>55 *vs* ≤55)	0.81	0.63–1.03	0.087			
HBsAg (positive *vs* negative)	1.16	0.83–1.61	0.369			
Tumor size, cm (>8 *vs* ≤8)	3.20	2.58–3.92	<0.001	1.78	1.36–2.40	<0.001
Tumor number (multiple *vs* single)	1.90	1.51–2.38	<0.001	1.12	0.78–1.49	0.423
Tumor differentiation (III–IV *vs* I–II)	2.91	2.27–3.65	<0.001	1.21	0.87–1.76	0.362
Vascular invasion (present *vs* absent)	3.02	2.29–3.96	<0.001	1.63	1.18–2.26	0.004
BCLC (B + C *vs* 0 + A)	2.70	2.13–3.41	<0.001	1.43	0.92–2.22	0.104
Recurrence (present *vs* absent)	1.22	0.94–1.61	0.117			
AFP, ng/ml (>20 *vs* ≤20)	1.38	1.07–1.81	0.014	1.02	0.78–1.31	0.826
ALT, U/L (>40 *vs* ≤40)	2.20	1.72–2.69	<0.001	1.28	1.07–1.83	0.052
SII, ×10^9^/L (>330 *vs* ≤330)	1.86	1.548–2.32	<0.001	1.27	0.97–1.69	0.071
ALRI (>25.2 *vs* ≤25.2)	2.13	1.71–2.70	<0.001	1.16	0.86–1.59	0.324
NγLR (>103.6 *vs* ≤103.6)	2.94	2.25–3.86	<0.001	1.65	1.20–2.23	0.002
Use of NAs (present *vs* absent)	0.52	0.28–0.80	<0.001	0.47	0.24–0.76	0.008
*Validation cohort*						
Gender (male *vs* female)	1.11	0.72–1.75	0.521			
Age, y (>55 *vs* ≤55)	0.82	0.59–1.15	0.246			
HBsAg (positive *vs* negative)	0.92	0.61–1.36	0.653			
Tumor size, cm (>8 *vs* ≤8)	3.26	2.41–4.31	<0.001	1.56	1.04–2.35	0.018
Tumor number (multiple *vs* single)	1.76	1.26–2.47	0.001	1.39	0.91–2.12	0.133
Tumor differentiation (III–IV *vs* I–II)	3.23	2.38–4.31	<0.001	1.37	0.86–2.18	0.185
Vascular invasion (present *vs* absent)	3.12	2.30–4.28	<0.001	1.49	0.96–2.34	0.078
BCLC (B + C *vs* 0 + A)	2.76	2.04–3.77	<0.001	1.41	0.86–2.31	0.171
Recurrence (present *vs* absent)	1.14	0.86–1.62	0.323			
AFP, ng/ml (>20 *vs* ≤20)	2.26	1.69–3.27	<0.001	1.46	1.09–2.07	0.086
ALT, U/L (>40 *vs* ≤40)	1.79	1.35–2.38	0.002	1.11	0.74–1.75	0.522
SII, ×10^9^/L (>330 *vs* ≤330)	1.49	1.13–2.01	0.006	0.98	0.69–1.37	0.703
ALRI (>25.2 *vs* ≤25.2)	1.76	1.29–2.38	0.001	1.19	0.81–1.76	0.335
NγLR (>103.6 *vs* ≤103.6)	2.89	2.05–4.12	<0.001	1.91	1.31–2.73	0.001
Use of NAs (present *vs* absent)	0.47	0.22–0.71	<0.001	0.36	0.18–0.59	0.040

Factors demonstrated to be statistically significant in univariate analysis were entered in the multivariate analysis with the Cox proportional hazards regression model (Table 3 and Supplementary Table S1). Elevated NγLR was associated with elevated risks for OS (hazard ratio [HR], 1.65; 95% CI, 1.20–2.23; *P* = 0.002) and PFS (HR, 1.42; 95% CI, 1.07–1.97; *P* = 0.021). Additionally, a tumour size of >8 cm was also identified as an independent predictor for OS (HR, 1.78; 95% CI, 1.36–2.40; *P* < 0.001) and PFS (HR, 1.58; 95% CI, 1.17–2.12; *P* = 0.003), while presence of vascular invasion (HR, 1.63; 95% CI, 1.18–2.26; *P* = 0.004) was an independent predictor for OS. Patients who used NAs as antiviral treatment had a better OS (HR, 0.47; 95% CI, 0.24–0.76; *P* = 0.008) in the multivariate model.

### Validating the prognostic value of NγLR in the validation cohort

We further evaluated whether NγLR maintained its prognostic value in another independent cohort. Similar to the results from the training cohort, patients with NγLR > 103.6 had a significantly shorter OS (median, 42.78 months; 95% CI, 38.63–46.94) and PFS (median, 31.41 months; 95% CI, 26.95–35.87) than patients with NγLR ≤ 103.6 (median OS, 64.85 months; 95% CI, 59.92–69.78; *P* < 0.0001; median PFS, 55.43 months; 95% CI, 48.61–62.25; *P* < 0.0001) (Fig. 3B and Supplementary Fig. S1B). The results of the univariate analyses were very similar between training and validation cohorts (Table 3 and Supplementary Table S1). In the multivariate analysis, NγLR > 103.6 remained an independent predictor for OS (HR, 1.91; 95% CI, 1.31–2.73; *P* = 0.001) and PFS (HR, 1.62; 95% CI, 1.10–2.44; *P* = 0.015).

### Early recurrence rate and further stratified NγLR in patients with HCC

The Kaplan–Meier curves also revealed that the NγLR > 103.6 group was associated with a higher early recurrence rate compared with the NγLR ≤ 103.6 group in the training (Fig. 3C, *P* < 0.0001) and validation cohorts (Fig. 3D, *P* = 0.007).

To further develop the NγLR index, this linear predictive index was then categorized into three different groups. In the training cohort, 95 (20.5%), 252 (54.4%), and 116 (25.1%) patients were placed in the low (NγLR ≤ 64), intermediate (64 < NγLR ≤ 370), and high (NγLR >370) risk groups, respectively, and the median OS times were 65.89 months (95% CI, 60.46–71.32), 47.66 months (95% CI, 43.88–51.44) and 27.29 months (95% CI, 22.41–32.16), respectively (Fig. 3E *, P* < 0.0001). The median PFS times were 60.34 months (95% CI, 53.62–67.06), 40.31 months (95% CI, 36.15–44.47), and 23.45 months (95% CI, 18.49–28.41) for patients in the low, intermediate, and high risk groups, respectively (see Supplementary Figure S1C *P* < 0.0001).

Applying the optimized NγLR index to the validation cohort, 53 (20.3%), 153 (58.6%), and 55 (21.1%) patients were in the NγLR ≤ 64, 64 < NγLR ≤ 370, and NγLR >370 groups, respectively. The mean OS times were 67.69 (95% CI, 61.32–74.05), 52.91 (95% CI, 48.55–57.27), and 30.88 (95% CI, 25.04–36.73) months for patients at low, intermediate, and high risk, respectively (Fig. 3F, *P* < 0.0001), while the median PFS times were 58.87 (95% CI, 49.60–68.14), 41.49 (95% CI, 36.29–46.71), and 19.67 (95% CI, 14.97–24.39) months, respectively (see Supplementary Figure S1D, *P* < 0.0001).

### Prognostic values of NγLR in patients with early HCC (BCLC stage 0 and A)

**Figure 4:**
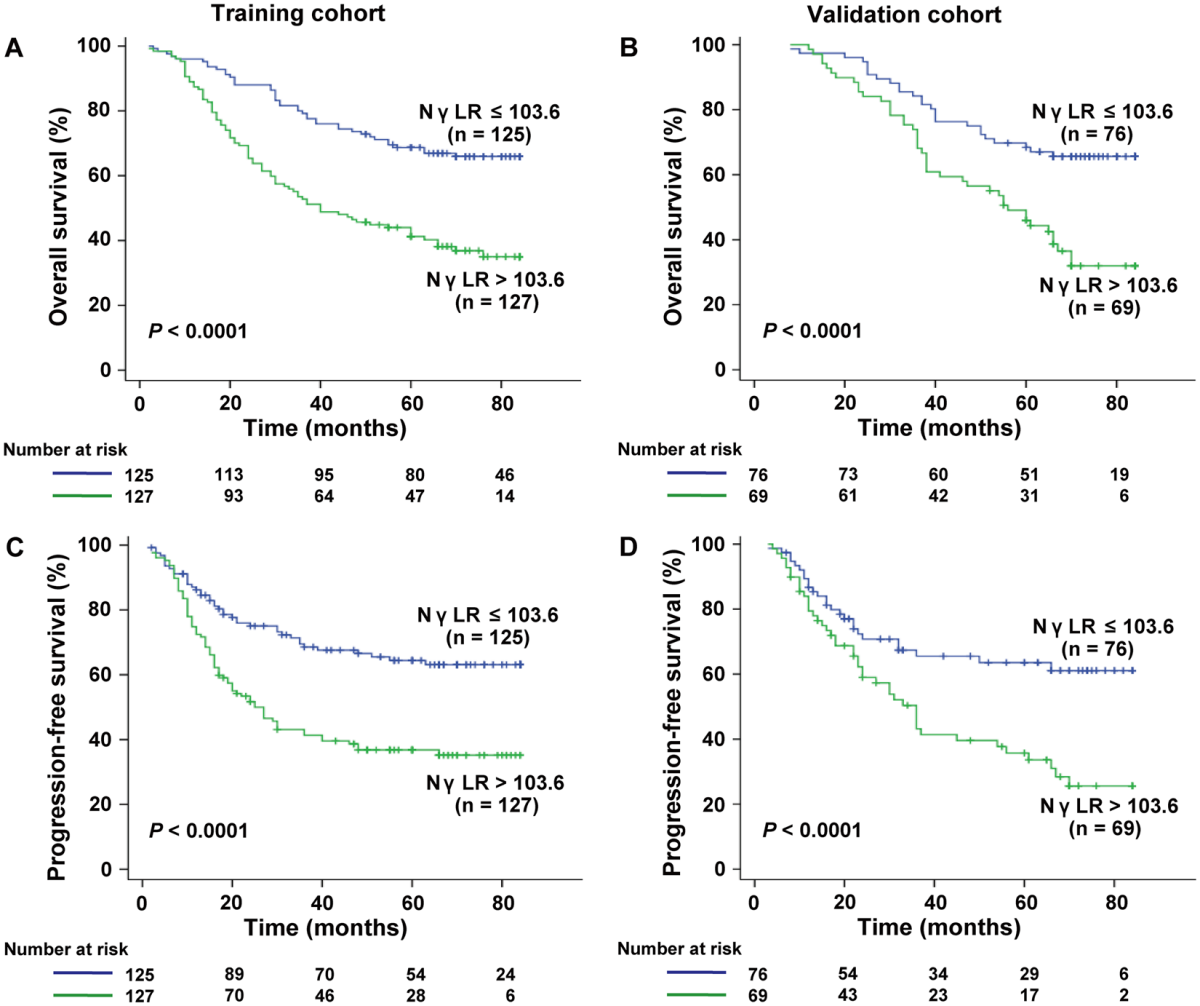
Prognostic significance of NγLR in early HCC patient. Kaplan-Meier curves depict overall survival (**A**,**B**) and progression-free survival (**C**,**D**) in early HCC patients (BCLC 0 + A) with NγLR > 103.6 or ≤103.6 in the training and validation cohorts.

In view of the prognostic values of NγLR in both training and validation groups, discriminative power of NγLR was further evaluated in early HCC (BCLC 0 + A) in greater detail. In the early-stage subgroup, NγLR > 103.6 was significantly associated with a shorter OS (median, 48.25 months; 95% CI, 42.94–53.55) and PFS (median, 41.48 months; 95% CI, 35.62–47.35) versus NγLR ≤ 103.6 (median OS, 66.54 months; 95% CI, 61.92-71.16; *P* < 0.0001; median PFS, 60.87 months; 95% CI, 55.09–66.64; *P* < 0.0001) in the training cohort (Fig. 4A and C). In the validation cohort, NγLR also significantly correlated with OS and PFS in the BCLC 0 + A subgroup (*P* < 0.0001 and *P* < 0.0001, respectively, Fig. 4B and D).

### Correlation between NγLR and BCLC stage

**Figure 5:**
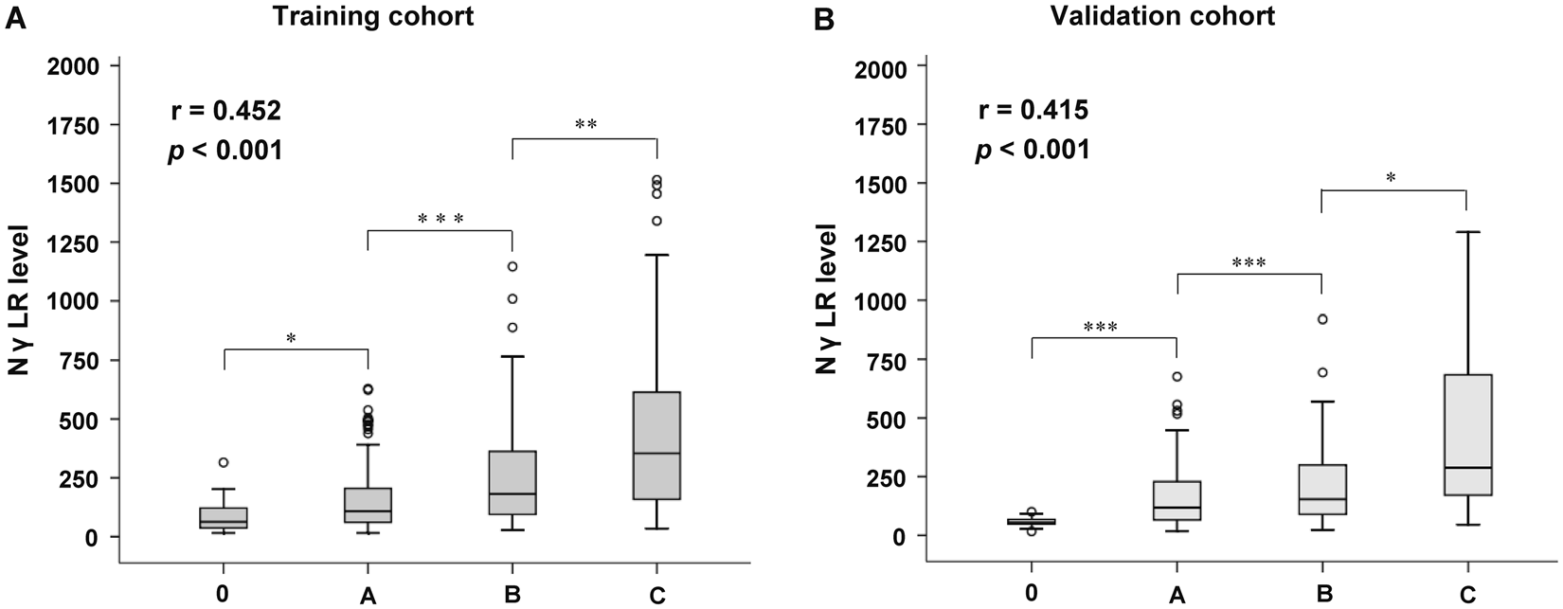
Box plots of NγLR values according to the BCLC stage in the training (A) and validation (B) cohorts. (*P < 0.05; **P < 0.01; ***P < 0.001)

BCLC stage is an important prognostic classification system for patients with HCC. Thus, we further analysed the relationship between the NγLR and the BCLC stage. Box plots of the NγLR in relation to the BCLC stage are presented in Fig. 5. In the training cohort, the severity of the BCLC stage was significantly positively correlated with a gradual increase in NγLR (r = 0.452, *P* < 0.001) (Fig. 5A). Furthermore, the results of the validation cohort (r = 0.415, *P* < 0.001) were the same as those obtained from the training cohort (Fig. 5B).

## Discussion

In this study, we established a simple and evidence-based prognostic index, namely, the NγLR, which incorporates routinely available laboratory parameters to predict the risk of recurrence and poor survival in HCC patients undergoing curative resection. This prognostic index was both accurate and reproducible. Patients in different subgroups of NγLR levels had distinctly different prognoses after surgery. As a linear risk index, NγLR further categorized patients into low, medium, and high risk groups with significantly different survival rates. The performance of the NγLR in predicting the risk stratification for prognosis was validated in another independent validation cohort with similar accuracy.

In the current study, the NγLR consisted of three factors: neutrophil count, lymphocyte count, and serum γ-GT level. Neutrophils and lymphocytes are peripheral blood cells that are associated with systemic inflammatory response and immune response. Neutrophils are the key players in inflammatory disorders, while lymphocytes are reflective of the host immune response. Therefore, an increase of NγLR may suggest activation of the inflammatory status and an immune-suppressive response in patients. Elevations of NγLR are usually associated with neutrophilia, elevated γ-GT concentration or lymphopenia. Neutrophils can promote the growth, invasion, and migration of cancer cells by releasing proinflammatory, immunoregulatory, and angiogenic factors^19–21^. Elevated γ-GT was also associated with mortality from many causes, including liver disease, cancer, and diabetes, even when controlling for alcohol consumption and restriction to mild and non-drinkers^22^. Activation of cytotoxic T lymphocytes could induce tumour killing by released cytokines, such as tumour necrosis factor-alpha and interferon-gamma. Meanwhile, endogenous T cells significantly delay malignant progression by responding to and infiltrating tumours^23^. Thus, it can be speculated that lymphopenia cannot effectively protect against the development of HCC by strengthening the body’s immune response. Accordingly, all of these were adverse factors for HCC patients. The NγLR integration of these three factors can better reflect their impact on HCC. In addition, our findings show that high NγLR was positively correlated with larger and multiple tumour number, poor differentiation, vascular invasion, severe BCLC stage, and early recurrence. It is postulated that NγLR represents a systemic inflammatory response; the elevation of NγLR may play an important role in maintaining the malignant phenotype of HCC cells and persistent damage of liver inflammation via internal environment disorders, thereby promoting the recurrence and metastasis of HCC. Our observation is consistent with a gene expression analysis that indicated that HCC metastases and relapse might be promoted by a shift towards anti-inflammatory/immune-suppressive responses^24^.

To date, AFP remains the most popular marker for the diagnosis and management of HCC. However, the sensitivity and specificity of AFP are limited; not all HCC patients secrete AFP. The sensitivity and specificity of AFP are only 61% and 81%, respectively, with a cut-off value of 20 ng/mL at the time of diagnosis^25^. Therefore, new markers are greatly needed to improve the ability to predict the prognosis of patients. In this study, we found that AUC for the NγLR was higher than that of AFP, and NγLR may be a novel prognostic marker for HCC patients. Moreover, our data showed that NγLR > 103.6 was also strongly correlated with the early recurrence of HCC. We know that the postoperative recurrence of HCC has two distinct mechanisms: the first is early recurrence via metastasis arising from undetected dissemination of tumour before surgery, and the other is late recurrence via de novo primary HCC in the liver remnant of dysplastic hepatocytes^26^. Postoperative recurrence, especially early recurrence, impacting the outcome of HCC is a very distressing situation for clinicians and patients. To date, no optimal marker has been identified. Although AFP is widely used in the postoperative monitoring of HCC, there are still 30% to 40% of HCC patients with normal serum AFP. Therefore, it is imperative to find other alternative predictor to compensate for this deficiency. Encouragingly, our study suggested that NγLR could serve as a new marker predicting early recurrence for postsurgical HCC patients. Accordingly, NγLR, as an inexpensive, objective, and readily available index, might be a promising marker of auxiliary diagnosis and postoperative recurrence monitoring for patients with HCC in clinical practice.

Previous studies have confirmed the prognostic values of SII^16^ and ALRI^17^ in HCC patients after surgery. The AUC of SII and ALRI to predict survival were lower than that of NγLR in this cohort; the same studies show inconsistent results, which may be associated with differences in the samples. Meanwhile, the prediction ability of NγLR was also higher than the conventional AFP. Therefore, NγLR had fairly good discriminatory power in stratifying patients with HCC into different prognostic groups.

It is widely accepted that BCLC stage is the most popular staging system with the power of prognostic stratification and therapeutic allocation^27, 28^. In this study, when we further explored the relationship between the NγLR and the BCLC stage, we found that the NγLR gradually increased with the increasing severity of BCLC stages. Meanwhile, the elevation of NγLR was associated with increasing risks of death and recurrence of HCC, and the NγLR had a fairly good prognostic power in both training and validation cohorts. Our study also demonstrated that NγLR still had a strong prognostic significance in early HCC patients. On the whole, our data indicated that NγLR could serve as a powerful prognostic marker for patients with HCC. The predictive significance of the NγLR in these subgroups is of great importance for clinicians to select the appropriate intervention after surgical resection to avoid unproven and futile treatment.

The NγLR was built in a cohort of patients with mainly HBV-related HCC. Effective antiviral therapy may potentially influence the prognosis of these patients. This finding is consistent with previously reported results that antiviral treatment is effective in a better survival^29^. However, antiviral therapy is prescribed in patients with a higher viral load and abnormal serum ALT levels, irrespective of cancer classification. This might explain why the predictive capability of NγLR may not be influenced by antiviral therapy.

The elevation of serum γ-GT concentration is strongly associated with heavy alcohol consumption. However, Ruhl *et al*.^22^ reported that a high relative risk of liver disease mortality with elevated γ-GT was not influenced by alcohol consumption. Therefore, alcohol consumption does not diminish the accuracy of the NγLR index in predicting prognosis of HCC. Moreover, HCC patients with alcohol consumption often stop drinking alcohol under doctors’ suggestions after being diagnosed with HCC.

We acknowledge that there are some limitations to our study. First, its retrospective nature is a potential limitation. Second, our study included HCC patients only from a single centre with a vast majority of patients having HBV infection. Although internally validated, whether our index can be generalized to different geographical areas remains to be determined. Third, the NγLR has been characterized in HCC patients undergoing curative resection; the prognostic ability of NγLR in palliative settings needs to be further explored in a future study.

In conclusion, we proposed a novel and simple prognostic index, the NγLR, including three readily available laboratory results for HCC patients after curative resection with a relative high degree of accuracy. The concept of the ratios among these three variables in the prediction of prognosis in patients with HCC is novel. NγLR can effectively identify the patients with HCC who are at the greatest risk of poor survival and early recurrence after surgery. It may be a useful tool for clinicians who manage postoperative patients with HCC. A prospective study to validate the NγLR index is being planned.

## Electronic supplementary material

Supplementary Information

## References

[1] Ferlay J. *et al*. GLOBOCAN 2012 v1.0, Cancer Incidence and Mortality Worldwide: IARC CancerBase No. 11 [Internet]. Lyon, France: International Agency for Research on Cancer; 2013. Available from: http://globocan.iarc.fr, accessed on 29/11/2016.

[2] El-Serag HB, Rudolph KL (2007). Hepatocellular carcinoma: epidemiology and molecular carcinogenesis. Gastroenterology.

[3] Llovet JM, Schwartz M, Mazzaferro V (2005). Resection and liver transplantation for hepatocellular carcinoma. Semin. Liver Dis..

[4] El-Serag HB (2011). Hepatocellular carcinoma. N. Engl. J. Med..

[5] A new prognostic system for hepatocellular carcinoma: a retrospective study of 435 patients: the Cancer of the Liver Italian Program (CLIP) investigators. *Hepatology* **28**, 751–755 (1998). 10.1002/hep.510280322 9731568

[6] Leung TW (2002). Construction of the Chinese University Prognostic Index for hepatocellular carcinoma and comparison with the TNM staging system, the Okuda staging system, and the Cancer of the Liver Italian Program staging system: a study based on 926 patients. Cancer.

[7] Tateishi R (2005). Proposal of a new prognostic model for hepatocellular carcinoma: an analysis of 403 patients. Gut.

[8] Yau T (2014). Development of Hong Kong Liver Cancer staging system with treatment stratification for patients with hepatocellular carcinoma. Gastroenterology.

[9] Camma C, Cabibbo G (2009). Prognostic scores for hepatocellular carcinoma: none is the winner. Liver Int..

[10] Zucman-Rossi J, Villanueva A, Nault JC, Llovet JM (2015). Genetic Landscape and Biomarkers of Hepatocellular Carcinoma. Gastroenterology.

[11] Villanueva A (2011). Combining clinical, pathology, and gene expression data to predict recurrence of hepatocellular carcinoma. Gastroenterology.

[12] Marquardt JU, Galle PR, Teufel A (2012). Molecular diagnosis and therapy of hepatocellular carcinoma (HCC): an emerging field for advanced technologies. J. Hepatol..

[13] Wong VW (2010). Clinical scoring system to predict hepatocellular carcinoma in chronic hepatitis B carriers. J. Clin. Oncol..

[14] Johnson PJ (2015). Assessment of liver function in patients with hepatocellular carcinoma: a new evidence-based approach-the ALBI grade. J. Clin. Oncol..

[15] Motomura T (2013). Neutrophil-lymphocyte ratio reflects hepatocellular carcinoma recurrence after liver transplantation via inflammatory microenvironment. J. Hepatol..

[16] Hu B (2014). Systemic immune-inflammation index predicts prognosis of patients after curative resection for hepatocellular carcinoma. Clin. Cancer Res..

[17] Jin J (2015). Elevated preoperative aspartate aminotransferase to lymphocyte ratio index as an independent prognostic factor for patients with hepatocellular carcinoma after hepatic resection. Oncotarget.

[18] Xu J (2012). An *in situ* molecular signature to predict early recurrence in hepatitis B virus-related hepatocellular carcinoma. J. Hepatol..

[19] Wislez M (2003). Hepatocyte growth factor production by neutrophils infiltrating bronchioloalveolar subtype pulmonary adenocarcinoma: role in tumor progression and death. Cancer Res..

[20] Queen MM, Ryan RE, Holzer RG, Keller-Peck CR, Jorcyk CL (2005). Breast cancer cells stimulate neutrophils to produce oncostatin M: potential implications for tumor progression. Cancer Res..

[21] Liang J (2014). Neutrophils promote the malignant glioma phenotype through S100A4. Clin. Cancer Res..

[22] Ruhl CE, Everhart JE (2009). Elevated serum alanine aminotransferase and gamma-glutamyltransferase and mortality in the United States population. Gastroenterology.

[23] DuPage M (2011). Endogenous T cell responses to antigens expressed in lung adenocarcinomas delay malignant tumor progression. Cancer cell.

[24] Budhu A (2006). Prediction of venous metastases, recurrence, and prognosis in hepatocellular carcinoma based on a unique immune response signature of the liver microenvironment. Cancer Cell..

[25] Lok AS (2010). Des-gamma-carboxy prothrombin and alpha-fetoprotein as biomarkers for the early detection of hepatocellular carcinoma. Gastroenterology.

[26] Imamura H (2003). Risk factors contributing to early and late phase intrahepatic recurrence of hepatocellular carcinoma after hepatectomy. J. Hepatol..

[27] Llovet JM (2008). Design and endpoints of clinical trials in hepatocellular carcinoma. J. Natl. Cancer Inst..

[28] Bruix, J., Sherman, M. & Practice Guidelines Committee, A. A. f. t. S. o. L. D. Management of hepatocellular carcinoma. *Hepatology* **42**, 1208-1236 (2005). 10.1002/hep.20933 16250051

[29] Wu CY (2012). Association between nucleoside analogues and risk of hepatitis B virus-related hepatocellular carcinoma recurrence following liver resection. Jama..

