# Supplementary material for: A novel prognostic index—neutrophil times γ-glutamyl transpeptidase to lymphocyte ratio (NγLR) predicts outcome for patients with hepatocellular carcinoma
Source: Sci Rep. 2017 Aug 23;7:9229. doi: 10.1038/s41598-017-09696-y (PMC5569032; doi:10.1038/s41598-017-09696-y)
Supplement: Supplementary file 1 — Supplementary Information [file 41598_2017_9696_MOESM1_ESM.doc]

**A** **novel prognostic index—neutrophil times γ-glutamyl transpeptidase to lymphocyte ratio (NγLR) predicts outcome for patients with hepatocellular carcinoma**

Jun Li, Yan Liao, Liya Suo, Pengpeng Zhu, Xinhuang Chen, Wei Dang, Minjun Liao, Liling Qin, Weijia Liao

**Contents:**

**Supplementary Table S1.** Analysis of progression-free survival in HCC patients in the training and validation cohort.

**Supplementary Figure S1. The relationship between NγLR and progression-free survival.** The Kaplan-Meier curves depict progression-free survival in HCC patients with NγLR > 103.6 or ≤ 103.6 (A-B) and according to the optimized NγLR (C-D) in training and validation cohorts.

| **Variable** | **Univariate analysis** | | | **Multivariate analysis** | | |
| --- | --- | --- | --- | --- | --- | --- |
|  | **HR** | **95% CI** | ***P* value** | **HR** | **95% CI** | ***P* value** |
| Training cohort |  |  |  |  |  |  |
| Gender (male *vs* female) | 1.27 | 0.92-1.90 | 0.063 |  |  |  |
| Age, y (> 55 *vs* ≤ 55) | 0.83 | 0.65-1.06 | 0.144 |  |  |  |
| HBsAg (positive *vs* negative) | 1.11 | 0.80-1.54 | 0.518 |  |  |  |
| Tumor size, cm (> 8 *vs* ≤ 8) | 2.92 | 2.31-3.66 | <0.001 | 1.58 | 1.17-2.12 | 0.003 |
| Tumor number (multiple *vs* single) | 1.90 | 1.51-2.39 | <0.001 | 1.14 | 0.83-1.59 | 0.407 |
| Tumor differentiation (III–IV *vs* I–II) | 2.65 | 2.10-3.34 | <0.001 | 1.22 | 0.86-1.72 | 0.216 |
| Vascular invasion (present *vs* absent) | 2.41 | 1.86-3.16 | <0.001 | 1.25 | 0.91-1.75 | 0.127 |
| BCLC (B+C *vs* 0+A) | 2.47 | 1.92-3.09 | <0.001 | 1.41 | 0.93-2.21 | 0.107 |
| AFP, ng/ml (> 20 *vs* ≤ 20) | 1.35 | 1.04-1.76 | 0.022 | 1.01 | 0.78-1.33 | 0.766 |
| ALT, U/L (> 40 *vs* ≤ 40) | 2.23 | 1.78-2.75 | <0.001 | 1.32 | 0.96-1.83 | 0.083 |
| SII,×10^9^/L (> 330 *vs* ≤ 330) | 1.68 | 1.33-2.13 | <0.001 | 1.22 | 0.94-1.58 | 0.130 |
| ALRI (> 25.2 *vs* ≤ 25.2) | 2.11 | 1.69-2.63 | <0.001 | 1.21 | 0.89-1.64 | 0.233 |
| NγLR (> 103.6 *vs* ≤ 103.6) | 2.59 | 1.99-3.41 | <0.001 | 1.42 | 1.07-1.97 | 0.021 |
| Use of NAs (present *vs* absent) | 0.51 | 0.32-0.83 | <0.001 | 0.45 | 0.27-0.73 | 0.062 |
| Validation cohort |  |  |  |  |  |  |
| Gender (male *vs* female) | 1.14 | 0.75-1.79 | 0.501 |  |  |  |
| Age, y (> 55 *vs* ≤ 55) | 0.92 | 0.69-1.28 | 0.516 |  |  |  |
| HBsAg (positive *vs* negative) | 1.06 | 0.73-1.51 | 0.722 |  |  |  |
| Tumor size, cm (> 8 *vs* ≤ 8) | 2.57 | 1.88-3.43 | <0.001 | 1.34 | 0.89-2.02 | 0.134 |
| Tumor number (multiple *vs* single) | 1.73 | 1.23-2.42 | 0.001 | 1.21 | 0.85-1.79 | 0.316 |
| Tumor differentiation (III–IV vs I–II) | 2.88 | 2.09-4.01 | <0.001 | 1.40 | 0.86-2.31 | 0.190 |
| Vascular invasion (present *vs* absent) | 2.70 | 1.97-3.66 | <0.001 | 1.38 | 0.89-2.15 | 0.133 |
| BCLC (B+C *vs* 0+A) | 2.62 | 1.91-3.54 | <0.001 | 1.49 | 1.03-2.35 | 0.031 |
| AFP, ng/ml (> 20 *vs* ≤ 20) | 2.32 | 1.67-3.25 | <0.001 | 1.30 | 1.04-2.09 | 0.086 |
| ALT, U/L (> 40 *vs* ≤ 40) | 1.72 | 1.26-2.34 | 0.001 | 0.77 | 0.52-1.13 | 0.072 |
| SII,×10^9^/L (> 330 *vs* ≤ 330) | 1.36 | 1.02-1.81 | 0.040 | 0.94 | 0.67-1.32 | 0.628 |
| ALRI (> 25.2 *vs* ≤ 25.2) | 1.72 | 1.27-2.34 | 0.001 | 1.38 | 0.94-2.04 | 0.099 |
| NγLR (> 103.6 *vs* ≤ 103.6) | 2.55 | 1.85-3.64 | <0.001 | 1.62 | 1.10-2.44 | 0.015 |
| Use of NAs (present *vs* absent) | 0.46 | 0.23-0.81 | <0.001 | 0.38 | 0.22-0.69 | 0.041 |

**Supplementary Table S1.** **Analysis of progression-free survival in HCC patients in the training and validation cohort.** HBsAg, hepatitis B surface antigen; BCLC, barcelona-clinic liver cancer; AFP, alpha-fetoprotein; ALT, alanine aminotransferase; SII, systemic immune-inflammation index; ALRI, aspartate transaminase to lymphocyte ratio index; NγLR, neutrophil cell count times γ-glutamyl transpeptidase to lymphocyte count ratio; NAs, nucleoside analogues.


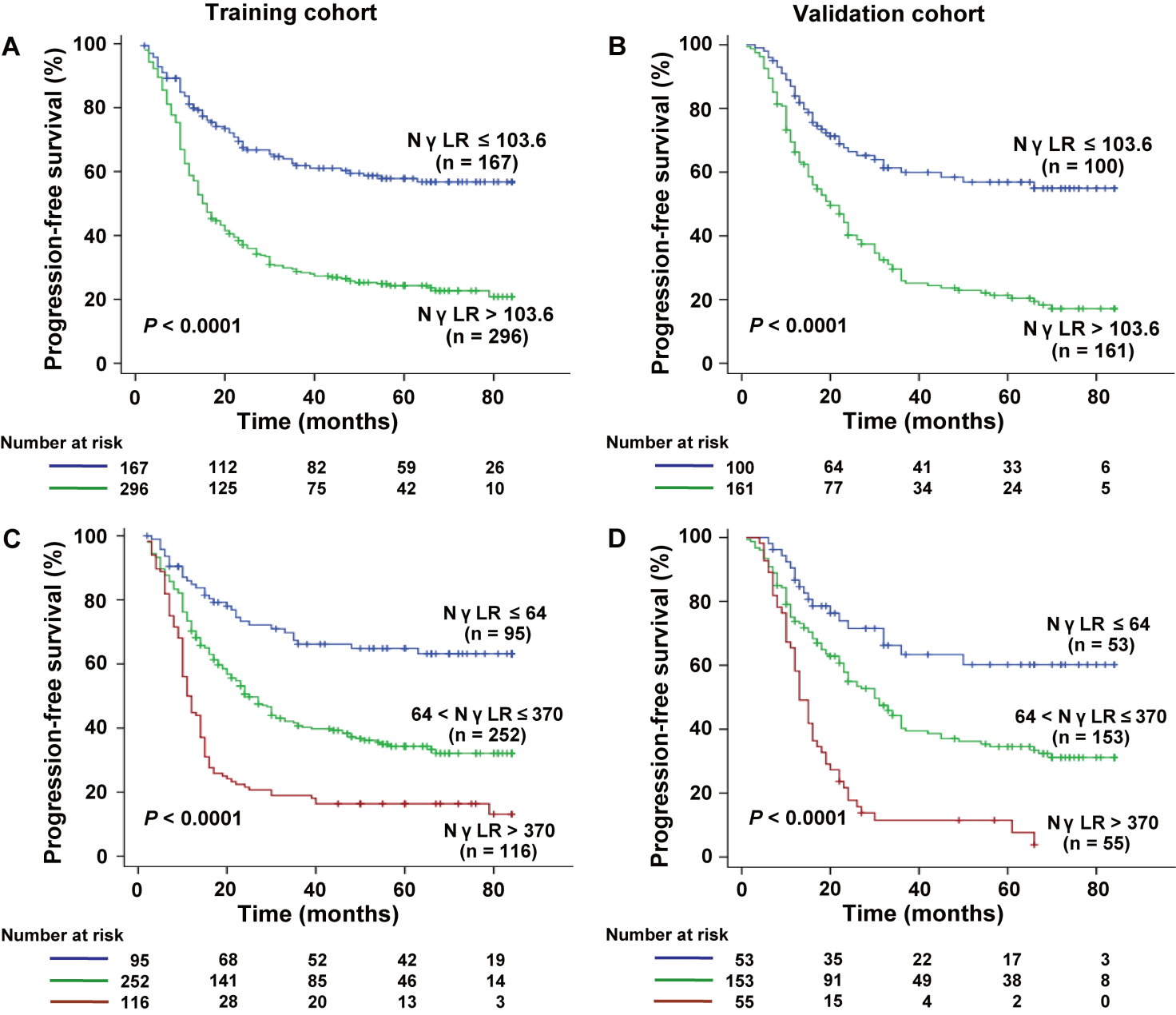


**Supplementary Figure S1. The relationship between NγLR and progression-free survival.** The Kaplan-Meier curves depict progression-free survival in HCC patients with NγLR > 103.6 or ≤ 103.6 (A-B) and according to the optimized NγLR (C-D) in training and validation cohorts.
